# Impact of Enhanced Recovery after Surgery^®^ Protocol Compliance on Length of Stay, Bowel Recovery and Complications after Radical Cystectomy

**DOI:** 10.3390/diagnostics14030264

**Published:** 2024-01-25

**Authors:** Nuno Grilo, François Crettenand, Perrine Bohner, Sonia Cristina Rodrigues Dias, Yannick Cerantola, Ilaria Lucca

**Affiliations:** Urology Department, Lausanne University Hospital, 1011 Lausanne, Switzerland

**Keywords:** ERAS^®^, enhanced recovery after surgery, compliance, bladder cancer, cystectomy

## Abstract

Background: Despite existing standardized surgical techniques and the development of new perioperative care protocols, radical cystectomy (RC) morbidity remains a serious challenge for urologists. Postoperative ileus (POI) is one of the most common postoperative complications, often leading to a longer length of stay (LOS). The aim of our study was to assess the impact of compliance to the Enhanced Recovery After Surgery (ERAS^®^) protocol on bowel recovery, 30-day complications and LOS after RC for bladder cancer (BC). Methods: Data from consecutive patients undergoing RC for BC within an ERAS^®^ dedicated protocol were analyzed. Exclusion criteria were urinary diversion other than ileal conduit and palliative RC. Patients were divided into two groups according to their compliance (A: low-compliance and B: high-compliance). ERAS^®^ compliance was extracted from the ERAS^®^ Interactive Audit System (EIAS) database. Postoperative complications were prospectively recorded by a dedicated study nurse 30 days after RC. POI was defined as the placement of a nasogastric tube. Logistic regression analysis was used to identify predictors of 30-day complications and POI. Results: After considering the exclusion criteria, 108 patients were included for the final analysis. The median global compliance to the ERAS^®^ protocol was 61%. A total of 78 (72%) patients had a compliance <65% (group A), while the remaining 30 (28%) had a compliance >65% (group B). No significant differences were found among the two groups regarding the 30-day complication rate (86% in group A versus 73% in group B, *p* = 0.82) and LOS (14 days in group A versus 15 days in group B, *p* = 0.82). The time to stool was significantly shorter in group B (4 days versus 6 days, *p* = 0.02), and the time to tolerate solid food was slightly faster in group B but not significant (8 versus 7 days, *p* = 0.23). The POI rate was significantly lower in patients with a higher ERAS^®^ compliance (20% versus 46%, *p* = 0.01). A multivariate analysis showed that ERAS^®^ compliance was not significantly associated with 30-day total complications. However, a lower compliance to the ERAS^®^ protocol and age > 75 years were significant independent predictors of POI. Conclusions: Our study provides further evidence to support the beneficial effect of the ERAS^®^ protocol in patients undergoing RC, particularly in terms of facilitating a faster recovery of bowel function and preventing POI. Future research should focus on investigating novel approaches and interventions to improve compliance with the ERAS^®^ protocol. This may involve patient education, multidisciplinary teamwork, and continuous quality improvement initiatives.

## 1. Introduction

Bladder cancer (BC) is recognized as the 10th most prevalent malignant tumor worldwide, and the 5-year cancer-specific survival rate in the United States stands at 77% [1]. Radical cystectomy (RC) with pelvic lymph node dissection is considered the gold standard treatment for recurrent high-risk non-muscle invasive BC (NMIBC) and muscle-invasive BC (MIBC). Despite significant advancements in surgical techniques, anesthesia, and perioperative care protocols, RC remains a complex and challenging procedure for urologists, often associated with considerable morbidity rates.

Postoperative ileus (POI) poses a considerable challenge following RC, frequently leading to additional complications, prolonged hospitalization, and increased readmission rates [2]. It is, therefore, associated with a significant negative economic impact on treatment-related costs [3]. The definition for POI is highly variable but is generally defined as diminished intestinal motility after surgery beyond the expected transient cessation of bowel activity. It commonly includes symptoms such as nausea and vomiting, intolerance of oral intake, bloating, abdominal pain, and other signs of bowel disfunction. The absence of accurate classification criteria makes it difficult to determine the exact incidence of POI. However, POI is estimated to be present in 10–40% of the patients undergoing RC and can contribute to a significant portion, accounting for 50–70%, of all postoperative complications [4,5,6,7]. Notably, no validated demographic or tumor characteristics, including gender, age or tumor stage, have emerged as reliable predictors of POI. To mitigate the risk of POI, strategies such as early postoperative mobilization, optimized pain management protocols with reduced reliance on opioids, and the administration of prokinetic agents to enhance bowel motility have been established as crucial supportive measures.

**Figure 1:**
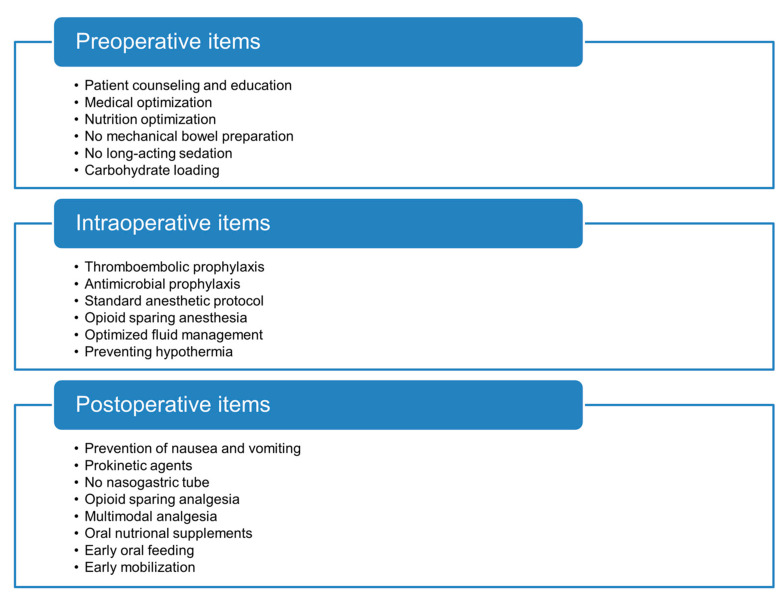
ERAS^®^ protocol key items.

Originally developed for colorectal surgery, the Enhanced Recovery After Surgery (ERAS^®^) protocol has emerged as a multimodal approach (Figure 1) aimed at expediting recovery and minimizing complications by optimizing perioperative care [8]. The ERAS^®^ protocol encompasses a comprehensive set of pre-, intra- and post-operative interventions and has become the standard of care.

However, achieving compliance with the protocol remains a critical factor in reducing surgical morbidity. Numerous research groups have shared their experiences in implementing ERAS^®^ protocols for RC patients. While the ERAS^®^ protocol has demonstrated improvements in bowel function recovery and reduced length of hospital stay without compromising readmission rates, its impact on reducing major complication rates has not yet reached statistical significance [9]. 

In light of the aforementioned considerations, the primary objective of our study was to assess the influence of compliance with the ERAS^®^ protocol on clinical outcomes, such as bowel recovery, 30-day complications and length of stay, among BC patients undergoing RC. Through this investigation, we aim to contribute to the growing body of evidence surrounding the use of ERAS^®^ protocols in urologic surgery and identify opportunities to optimize compliance, ultimately enhancing patient outcomes. By expanding our understanding of the impact of ERAS^®^ implementation in the context of RC, we can provide valuable insights for healthcare professionals and guide further advancements in perioperative care.

## 2. Materials and Methods

### 2.1. Study Population

Following institutional review board approval, we conducted a retrospective analysis of data from consecutive patients who underwent RC for BC at Lausanne University Hospital between 2012 and 2020. 

**Figure 2:**
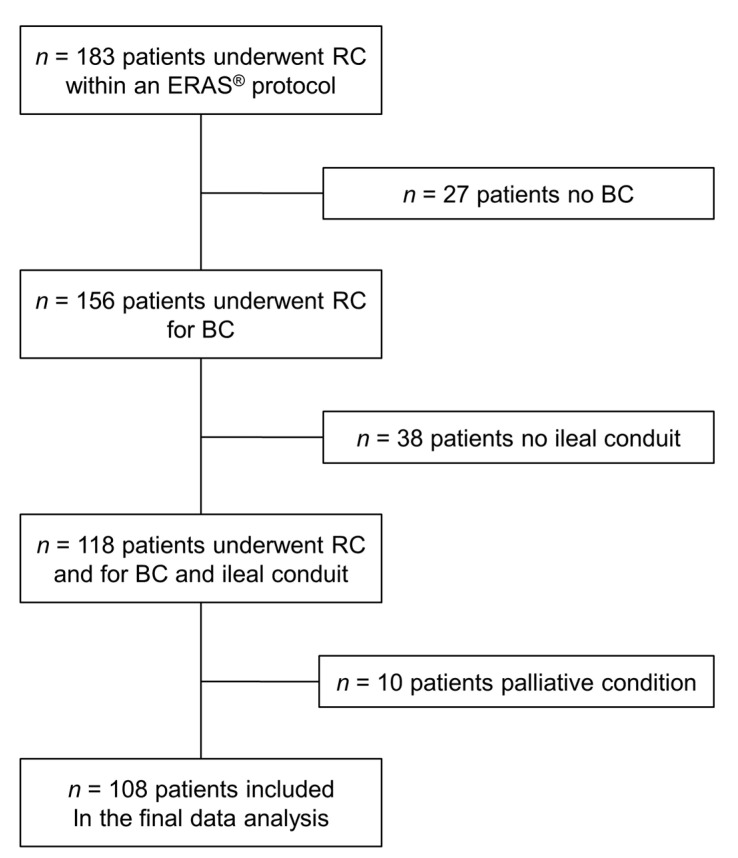
Study flow chart, with counts and reasons for patient selection.

The surgical procedures were performed by a consistent team of skilled bladder cancer surgeons who adhered to an internally standardized protocol, ensuring uniformity in the surgical approach. All surgeries were performed by three senior surgeons skilled in RC for BC, and all of them were trained with the same surgical technique. The same BC team performed all surgeries regardless of the patients’ group. Extended lymphadenectomy (external, internal and common iliac vessels, obturator fossa, Marcille fossa) was performed in all patients. To enhance the homogeneity of our study population, we applied specific exclusion criteria, removing patients with urinary diversion methods other than ileal conduit (IC) and those with palliative cases (Figure 2). This selection process resulted in a final cohort comprising 108 patients who met the inclusion criteria. To further analyze the impact of compliance with the ERAS^®^ protocol, we categorized the patients into two distinct groups: Group A consisted of individuals with a compliance rate lower than 65% (*n* = 78), while Group B comprised patients who exhibited a compliance rate higher than 65% (*n* = 30).

### 2.2. ERAS^®^ Protocol and Data Collection

All patients enrolled in this study strictly adhered to an ERAS^®^ protocol specifically designed for RC, following the guidelines recommended by the ERAS^®^ society [10]. To ensure meticulous data collection and monitoring, a dedicated study nurse prospectively recorded various pre-, intra- and postoperative variables using the ERAS^®^ Interactive Audit System (EIAS), which underwent regular quality assessments conducted by the ERAS^®^ society. The variables analyzed encompassed demographic information such as age, sex and American Society of Anesthesiologists (ASA) score, as well as other relevant factors including smoking status, preoperative chemotherapy and final histopathology outcomes including T and N tumor stage. Furthermore, intraoperative factors such as operative time and blood loss were diligently evaluated. Postoperatively, complications were recorded during the 30-day follow-up period, with severity assessments based on the established Clavien–Dindo classification system, classifying complications as minor (Clavien–Dindo I-II) or major (Clavien–Dindo III-V). Of particular interest, the occurrence of POI was defined as the necessity for nasogastric tube placement. Additional data points collected included the length of hospital stay (LOS), the time required to tolerate solid food (defined as the number of days until two consecutive solid foods could be tolerated), the time to the first defecation (number of days to pass formed stools), as well as the 30-day reoperation and readmission rates. Compliance with the ERAS^®^ protocol was meticulously extracted from the EIAS database. However, preadmission compliance items were excluded from our analysis due to inherent heterogeneity within the data. The implementation of changes in the EIAS system in 2015 introduced significant heterogeneity, resulting in artificially low compliance rates for preadmission items.

### 2.3. Statistical Analysis

The categorical variables were presented as numbers and percentages, providing valuable insights into the distribution patterns of various factors within the study population. On the other hand, continuous variables were expressed using median values and the interquartile range (IQR), offering a robust representation of the central tendency and dispersion of the data. To explore the potential associations and differences between groups, comprehensive statistical analyses were conducted. For categorical variables, chi-square tests were employed, allowing for the evaluation of group differences and determining the significance of associations. Similarly, for continuous variables, the Kruskal–Wallis test was utilized, enabling the comparison of group medians and assessing potential variations across the groups.

To delve deeper into the factors influencing the occurrence of 30-day complications, POI and LOS, both univariable and multivariable logistic regression analyses were performed. The univariable analysis provided a preliminary examination of the individual associations between each variable and the respective outcome measures. Variables that demonstrated statistical significance in the univariable analysis were then included in the multivariable analysis, which employed a more comprehensive model to identify the independent predictors of the outcomes of interest. Inclusion in the multivariable analysis was based on a *p*-value threshold of less than 0.05, indicating statistical significance.

All statistical analyses were conducted using the STATA 15 software package (College Station, TX, USA).

## 3. Results

### 3.1. Patient Characteristics and Postoperative Outcomes

Clinical and pathological characteristics are shown in Table 1. The analysis included a total of 108 patients, with 77 (71%) being male and a median age of 73 years (IQR 67–77). Preoperative chemotherapy was administered to 20 patients (18%). Among the study cohort, 78 patients (72%) exhibited a compliance rate with the ERAS^®^ protocol lower than 65% (Group A). Baseline characteristics, such as age, sex, ASA score, smoking status, preoperative chemotherapy and tumor stage, were similar between Group A (compliance lower than 65%, *n* = 78) and Group B (compliance higher than 65%, *n* = 30).

Regarding postoperative outcomes, Group B presented a significantly shorter time to stool compared to Group A, with a median of 4 days versus 6 days, respectively (*p* = 0.02). Although not statistically significant, Group B also exhibited a slightly shorter time to tolerate solid food, with a median of 8 days compared to 7 days in Group A (*p* = 0.23). The overall 30-day complication rate did not significantly differ between the groups, with rates of 86% in Group A and 73% in Group B (*p* = 0.12). Furthermore, there were no significant differences in major complication rates, as evidenced by rates of 31% in Group A and 33% in Group B (*p* = 0.42). An interesting finding emerged in relation to POI, with a significantly lower rate observed in patients who exhibited a higher compliance with the ERAS^®^ protocol. In Group B, the rate of POI was 20%, whereas in Group A, it was 46% (*p* = 0.01). These results suggest that greater adherence to the ERAS^®^ protocol is associated with a reduced risk of POI in patients undergoing RC. The LOS did not significantly differ between the groups, with a median LOS of 14 days in Group A and 15 days in Group B (*p* = 0.82). Similarly, the readmission rate within 30 days did not exhibit a significant difference, with rates of 17% in Group A and 20% in Group B (*p* = 0.79).

### 3.2. Compliance to ERAS^®^ Protocol

Compliance to ERAS^®^ protocol is presented in Table 2. Postoperative items, such as early mobilization, early oral energy supplements intake and early termination of IV fluids had the lowest compliance, while preoperative items (oral mechanical bowel preparation, preoperative carbohydrate loading, preoperative long-acting sedation, thrombosis prophylaxis and antimicrobial prophylaxis) had the highest compliance between 92% and 100%.

### 3.3. Univariable and Multivariable Analyses

ERAS^®^ compliance was not significantly associated with a 30-day overall complication rate. In univariable analysis, only ASA score (OR 7.95, 95%CI 1.73–36.43, *p* = 0.008) was significantly associated with a higher risk of complication at 30 days (Table 3A). However, in multivariate analysis, compliance to the ERAS^®^ protocol (OR 0.27, 95%CI 0.10–0.76, *p* = 0.01) and age >75 years (OR 2.51, 95%CI 1.09–5.74, *p* = 0.03) were both significant independent predictors of POI (Table 3B).

## 4. Discussion

The present study aimed to comprehensively evaluate the impact of the compliance with the ERAS^®^ protocol on bowel recovery, 30-day complications and length of stay, in patients undergoing RC for BC. Patients with higher compliance to the ERAS^®^ protocol exhibited a significantly shorter time to stool compared to those in the lower compliance group. Furthermore, there was a notable trend towards a shorter time to tolerate solid food. These compelling findings are consistent with previous studies that have consistently demonstrated a faster recovery of bowel function with the use of the ERAS^®^ protocol in colorectal surgeries [11,12]. 

Our results elucidated that compliance with the ERAS^®^ protocol was suboptimal for specific postoperative items, including early mobilization, early oral energy supplement intake and early termination of intravenous (IV) fluids. It is worth noting that similar findings have been reported in other studies within the colorectal surgery field [13,14]. In our experience, we encountered significant challenges in achieving early mobilization and early oral intake in BC patients undergoing RC, particularly in those with severe comorbidities and compromised postoperative status.

Higher compliance with the ERAS^®^ protocol has shown a positive impact on reducing the LOS in patients undergoing RC [15]. However, in our series, the compliance rate with the ERAS^®^ protocol did not exhibit a significant influence on the duration of hospitalization. This intriguing finding might be attributed to several factors. For instance, group and individual cultural factors may have influenced patient behavior and expectations regarding hospital discharge. Moreover, the practice of discharging a limited number of patients to rehabilitation facilities may have introduced bias, as patients often have to await a vacancy before they can be discharged.

Concerning the overall complication rate, it is widely acknowledged that RC is a major urological procedure associated with a high morbidity. Notably, a recent meta-analysis evaluating short-term morbidity following RC for BC revealed an average 30-day complication rate of 39%, with a rate of major complications reaching 15.5% [16]. Interestingly, our cohort exhibited a higher 30-day complication rate, which might be attributed to the prospective data collection method and the specific definitions employed in our study. POI emerged as the most prevalent complication, observed in 39% of the patients. Of particular interest is the robust association between ERAS^®^ compliance and the incidence of POI. Our study unveiled that compliance to the ERAS^®^ protocol serves as an independent predictor of POI, as patients with lower compliance exhibited a significantly higher rate of POI compared to those with higher compliance. These findings underscore the critical role played by adherence to various components of the ERAS^®^ protocol, such as early mobilization, early oral energy supplement intake and early termination of IV fluids, in mitigating the risk of POI. Importantly, this conclusion aligns with the results of a comprehensive systematic review and meta-analysis, which established a significant reduction in POI with higher compliance to the ERAS^®^ protocol in patients undergoing colorectal surgery [17]. Furthermore, our study found that age > 75 years was another significant independent predictor of a higher risk for POI. This observation is consistent with a nationwide analysis of more than 40,000 patients undergoing RC in the United States [18] and another prospective study including 2538 patients [7], which also found age as an independent risk factor for POI. Hemorrhage emerged as the second most common complication, affecting 35% of the patients. For the purpose of this study, hemorrhage was defined as the need for receiving two or more blood units intraoperatively or any blood unit within the subsequent 48 h. It is important to note that this finding is often not included as a complication in the majority of studies. Given the critically ill state of a substantial proportion of BC patients at the time of RC and the prevalence of chronic anemia, the observed transfusion rate during and after major surgery is unsurprising in this specific population [19]. 

With regard to the impact of compliance to the ERAS^®^ protocol on the overall complication rate, our study did not identify a significant difference between the two groups. Intriguingly, the ASA score ≥ 3 emerged as the sole predictor of the overall complication rate. Unsurprisingly, this result aligns with a prior study that also identified the ASA score as an independent predictor of overall complications in patients undergoing RC. Notably, surgical high-risk patients with ASA 3 to 4 undergoing RC may face double the mortality and morbidity rates compared to individuals with ASA 1 to 2 [20]. 

In terms of the readmission rate within 30 days, our study yielded no significant difference between the two groups. This finding is consistent with numerous other studies that have consistently demonstrated no significant difference in readmission rates between ERAS^®^ and conventional care in patients undergoing RC [21,22,23].

While our study found some promising results regarding the effectiveness of the ERAS^®^ protocol in patients undergoing RC, there are several limitations that should be considered. Firstly, our study was conducted as a single-center retrospective analysis, which may limit the generalizability of our findings to other settings. However, it is important to note that our data were prospectively recorded in the dedicated EIAS database, following standardized protocols, and underwent independent data verification, enhancing the quality of the data. The relatively small sample size is a major limitation and may reduce the statistical power of our study and increases the risk of type II errors. 

The long study period also presents a limitation, as it implies that the data might not fully represent current practice patterns. Over time, the ERAS^®^ protocols have been updated and refined, which may have influenced the outcomes observed in our study. Additionally, the definition of postoperative ileus (POI) in our study was based on the placement of a nasogastric tube. However, it is worth noting that there is no universally validated definition of POI, which makes it challenging to compare our findings with other studies using different criteria. Moreover, our investigation focused exclusively on open RC, and the impact of ERAS^®^ compliance on POI, LOS and 30-day complications in minimally invasive surgical approaches was not explored. Therefore, the generalizability of our findings to these surgical techniques is limited. 

Despite these limitations, our study provides valuable insights into the use of ERAS^®^ protocols in urologic surgery and underscores the importance of ongoing efforts to improve compliance and optimize patient outcomes. Further research with larger sample sizes, multi-center designs and a standardized definition of POI is warranted to validate and expand upon our findings.

## 5. Conclusions

Our study provides further evidence to support the beneficial effect of the ERAS^®^ protocol in patients undergoing RC, particularly in terms of facilitating a faster recovery of bowel function and preventing POI. Future research should focus on investigating novel approaches and interventions to improve compliance with the ERAS^®^ protocol. This may involve patient education, multidisciplinary teamwork and continuous quality improvement initiatives.

## Figures and Tables

**Table 1:** Patients’ characteristics.

Variable	All Patients	ERAS^®^ Compliance < 65	ERAS^®^ Compliance > 65	*p*-Value
*n* (%)	108	78 (72)	30 (28)	
Age—median (IQR)	73 (67–77)	77 (76–78)	73 (60–80)	0.62
Gender—*n* (%)				0.44
Female	31 (29)	23 (29)	7 (23)	
Male	77 (71)	54 (69)	23 (77)	
Smoking—*n* (%)	41 (38)	28 (36)	13 (43)	0.51
Diabetes—*n* (%)	20 (19)	14 (18)	6 (20)	0.80
Charlson—median (IQR)	6 (5–7)	6 (5–8)	5.5 (4–7)	0.31
Previous radiotherapy to op. field—*n* (%)	6 (6)	5 (6)	1 (4)	0.53
Previous surgery to op. field—*n* (%)	51 (47)	36 (46)	15 (50)	0.76
Preoperative chemotherapy—*n* (%)	20 (18)	12 (15)	8 (27)	0.17
Tumor stage—*n* (%)				0.71
pTa-T1-Tis	38 (36)	25 (32)	13 (43)	
pT2	14 (13)	11 (14)	3 (10)	
pT3	38 (35)	29 (37)	9 (30)	
pT4	18 (16)	13 (17)	5 (17)	
pN stage—*n* (%)				0.91
pN0	80 (74)	58 (74)	22 (73)	
pN+	28 (26)	20 (26)	8 (27)	
ASA score				0.28
1–2	63 (58)	43 (55)	20 (67)	
3–4	45 (42)	35 (45)	10 (33)	
Complications—*n* (%)				
Total	89 (82)	67 (86)	22 (73)	0.12
Minor	55 (51)	43 (55)	12 (40)	
Major	34 (31)	24 (31)	10 (33)	0.42
Postoperative Ileus	42 (39)	36 (46)	6 (20)	0.01
Operation time—median (IQR)	371 (335–425)	371 (337–417)	370.5 (327–432)	0.98
Blood loss—mL, median (IQR)	700 (500–1000)	725 (500–1000)	600 (450–950)	0.30
LOS—median (IQR)	15 (12–21)	14 (12–23)	15 (12–20)	0.82
Time to stool—days, median (IQR)	5 (4–7)	6 (4–8)	4 (3–6)	0.02
Time to solid food—days, median (IQR)	8 (5–12)	8 (5–14)	7 (5–8)	0.23
Readmission—*n* (%)	19 (18)	13 (17)	6 (20)	0.79
Reoperation—*n* (%)	24 (22)	17 (22)	7 (23)	0.83

**Table 2:** Compliance to ERAS^®^ protocol.

ERAS^®^ Single Item	Compliance (%)
Oral mechanical bowel preparation (NO)	97
Preoperative carbohydrate loading (YES)	99
Preoperative long-acting sedation (NO)	92
Thrombosis prophylaxis (YES)	99
Antimicrobial prophylaxis (YES)	100
Prevention of postoperative nausea and vomiting (YES)	97
Systemic opioids (NO)	56
Preventing intraoperative hypothermia (YES)	100
Nasogastric intubation (NO)	82
Stimulation of gut motility (YES)	99
Multimodal postoperative analgesia (YES)	92
Termination of IV fluids by POD 5 (YES)	56
Oral energy supplements intake on POD 1 (>300 kcal) (YES)	49
Mobilization on POD 1 (>3 h out of bed) (YES)	37
Mobilization on POD 2 (>3 h out of bed) (YES)	37
Mobilization on POD 3 (>6 h out of bed) (YES)	21
Total	61

**Table 3:** (**A**). Univariate analysis predicting 30 day complications. (**B**). Univariable and multivariable analysis predicting POI.

(**A**)						
**Variable**	**Univariate**					
	**OR**	**95%CI**	***p***			
Age > 75 years	1.16	0.42–3.24	0.77			
BMI	0.99	0.90–1.10	0.97			
ASA score	7.95	1.73–36.43	0.008			
Operative time	1.01	0.99–1.01	0.10			
Blood loss > 600 cc	2.49	0.89–6.99	0.08			
ERAS^®^ compliance > 65%	0.45	0.16–1.26	0.13			
(**B**)						
**Variable**	**Univariate**			**Multivariate**		
	**OR**	**95%CI**	***p***	**OR**	**95%CI**	***p***
Age > 75 years	1.55	1.06–5.23	0.03	2.51	1.09–5.74	0.03
ASA score	1.27	0.58–2.78	0.55			
Operative time	0.99	0.99–1.01	0.55			
IV fluids > 4 cc/kg/h	1.98	0.20–19.73	0.56			
Blood loss > 600 cc	1.33	0.59–3.03	0.49			
48h postoperative opioids use	1.90	0.74–4.89	0.18			
ERAS^®^ compliance > 65%	0.29	0.11–0.79	0.02	0.27	0.10–0.76	0.01

## Data Availability

The datasets generated during and/or analyzed during the current study are available from the corresponding author on reasonable request.
